# Everolimus Based Immunosuppression Strategies in Adult Lung Transplant Recipients: Calcineurin Inhibitor Minimization Versus Calcineurin Inhibitor Elimination

**DOI:** 10.3389/ti.2023.10704

**Published:** 2023-01-20

**Authors:** Steven Ivulich, Eldho Paul, Carl Kirkpatrick, Michael Dooley, Greg Snell

**Affiliations:** ^1^ The Alfred Hospital, Melbourne, VIC, Australia; ^2^ Centre for Medication Use and Safety, Monash University, Melbourne, VIC, Australia; ^3^ Public Health and Preventative Medicine, Monash University, Melbourne, VIC, Australia

**Keywords:** lung transplantation, everolimus, calcineurin inhibitor, lung transplant recipients, mammalian-target-of-rapamycin inhibitor, lung transplant survival, nephrotoxicity

## Abstract

Everolimus (EVE) provides an alternative to maintenance immunosuppression when conventional immunosuppression cannot be tolerated. EVE can be utilized with a calcineurin inhibitor (CNI) minimization or elimination strategy. To date, clinical studies investigating EVE after lung transplant (LTx) have primarily focused on the minimization strategy to preserve renal function. The primary aim was to determine the preferred method of EVE utilization for lung transplant recipients (LTR). To undertake this aim, we compared the safety and efficacy outcomes of EVE as part of minimization and elimination immunosuppressant regimens. Single center retrospective study of 217 LTR initiated on EVE (120 CNI minimization and 97 CNI elimination). Survival outcomes were calculated from the date of EVE commencement. On multivariate analysis, LTR who received EVE as part of the CNI elimination strategy had poorer survival outcomes compared to the CNI minimization strategy [HR 1.61, 95% CI: 1.11–2.32, *p*=0.010]. Utilization of EVE for renal preservation was associated with improved survival compared to other indications [HR 0.64, 95% CI: 0.42–0.97, *p*=0.032]. EVE can be successfully utilized for maintenance immunosuppression post LTx, particularly for renal preservation. However, immunosuppressive regimens containing low dose CNI had superior survival outcomes, highlighting the importance of retaining a CNI wherever possible.

## Graphical Abstract

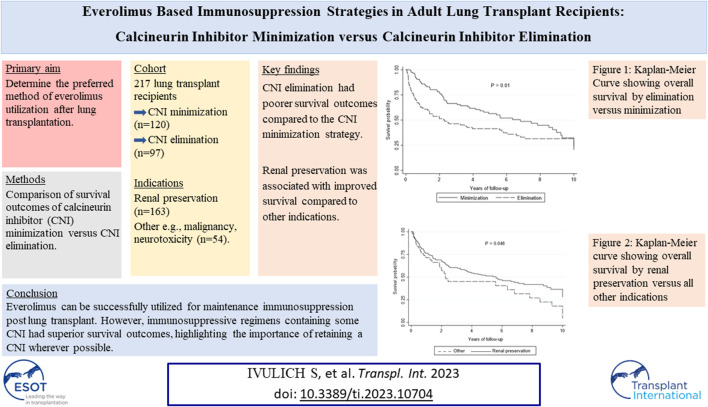

## Introduction

Maintenance immunosuppressant regimens after lung transplantation (LTx) typically consist of a calcineurin inhibitor (CNI, tacrolimus or ciclosporin), an antiproliferative (mycophenolate or azathioprine) and a corticosteroid (1). Immunosuppressant regimens protect against the development of chronic lung allograft rejection (CLAD), the main barrier preventing better long-term outcomes (2).

CNIs remain the cornerstone of immunosuppression post LTx to prevent allograft rejection, but their usage is limited by several side effects, predominantly nephron- and neurotoxicity. Antiproliferative immunosuppressants are associated with leukopenia, gastrointestinal side effects and hepatotoxicity (3, 4), and prednisolone is associated with diabetes mellitus and osteoporosis (5). Everolimus (EVE) provides a useful alternative when conventional maintenance regimens cannot be tolerated.

EVE has unique pharmacological effects distinct from other immunosuppressants. EVE may reduce the risk of malignancy or *Cytomegalovirus* (CMV) infection (6). However, EVE has its own limitations in the LTx setting with its use not recommended early post-LTx due to the risk of wound dehiscence and an association with pneumonitis, proteinuria, non-healing wounds, hematological and metabolic side effects (hyperlipidemia) (7).

The proportion of LTR not taking a CNI is rare with the International Society for Heart and Lung Transplantation (ISHLT) estimating that approximately 99% of lung transplant recipients (LTR) are taking a CNI at time of 1-year follow up (8). Reducing CNI exposure is a key component to reducing long-term CNI toxicity. To date, clinical studies investigating EVE after LTx have primarily focused on the minimization strategy to preserve renal function (9–13), with a paucity of evidence for elimination or other indications (e.g., malignancy, neurotoxicity). Most trials so far have demonstrated high rates of discontinuation, patient intolerance and significant side effects (9–11).

Given the limited evidence for CNI elimination after LTx and the implausibility of a randomized controlled trial, the primary aim of this study was to determine the preferred method of EVE utilization for LTR. To undertake this aim, we retrospectively compared the safety and efficacy outcomes of EVE as part of minimization and elimination immunosuppressant regimens.

## Materials and Methods

Between 2008 and 2020, 1,300 LTx were undertaken. In the current study, all recipients received standard triple immunosuppression with tacrolimus, an antimetabolite (azathioprine or mycophenolate mofetil) and corticosteroids. Ciclosporin was utilized as a second line CNI in settings where tacrolimus was withdrawn (e.g., CNI-neurotoxicity), but where the inclusion of a CNI was considered essential to maintain adequate immunosuppression. All individuals prescribed EVE were considered for inclusion with EVE being prescribed as a second line agent in 240 (18.5%). Excluded were those: lost to follow up, early discontinuation (duration of therapy <3 months), incomplete medical records or previous sirolimus therapy.

### Therapeutic Drug Monitoring (TDM)

Therapeutic Drug Monitoring (TDM) for ciclosporin and tacrolimus trough concentrations were obtained using ACQUITY Ultraperformance Liquid Chromatography (Waters Corporation, Manchester, United Kingdom). Ciclosporin trough concentration targets were 225–300 ng/mL in the first 3 months, 190–260 ng/mL between 3–12 months, and 150–225 ng/mL thereafter. Tacrolimus trough concentration targets were 10–12 ng/mL in first 6 months, 8–10 ng/mL between 6–12 months, and 4–8 ng/mL thereafter. Ciclosporin and tacrolimus targets were clinically modified in the presence of rejection episodes, significant renal impairment, or systemic sepsis (1, 14).

### EVE Indications, Dosing, and Utilization Strategy

EVE was utilized for clinical LTx where the traditional initial immunosuppressive strategies were contraindicated (e.g., significant renal impairment, CNI-neurotoxicity, malignancy) (1, 14). Allocation to either strategy was determined by the degree of CNI intolerance at the time of initiation of EVE, and the decision to withdraw or reduce the CNI was based on clinical judgement. As per unit protocol, EVE was typically commenced at a moderate dose of 0.25–0.5 mg twice daily, with the CNI dose immediately halved (15). The dose was subsequently adjusted according to the target level for the strategy utilized.

All included LTR were subsequently divided into two groups: CNI minimization (Group A) or CNI elimination (Group B). For CNI minimization, when EVE was to be used in conjunction with a CNI (Group A), an EVE trough serum concentration of 3–5 ng/mL was targeted. If CNI cessation was planned, an EVE trough serum concentration of 5–7 ng/mL was targeted and the CNI was ceased once the EVE trough level was >3 ng/mL.

If a LTR remained on a CNI following introduction of EVE (Group A) for renal preservation and measured serum creatinine concentrations continued to increase or remained high, they would then typically transition to Group B and the CNI withdrawn. LTR who withdrew from a CNI within 90 days post EVE introduction were included as CNI elimination. Concomitant medication with azathioprine or mycophenolate plus prednisolone were continued according to local practice.

### Donor Assessment, Recipient Selection, Transplantation Procedures and Postoperative Management

Our Alfred approach to lung donor referral, assessment and transplantation is described elsewhere (16, 17). Recipient selection is based on International and National Guidelines (18). Donor-recipient matching was generally undertaken according to our standard protocol as described elsewhere (19, 20). All patients received prophylactic antibiotics based on known or suspected donor and recipient microbiology results. CMV prophylaxis, monitoring and treatment strategies are described elsewhere (21). Surveillance bronchoscopy and transbronchial biopsies were performed as per hospital protocol at 2, 6-, 12-, 26- and 52-week post LTx.

### Definition of Rejection

Acute cellular rejection (ACR) was defined as changes on transbronchial biopsy of ≥ ISHLT Grade 2, or in the absence of a biopsy an otherwise unexplained drop in lung function treated with intravenous corticosteroid (14, 22). CLAD was clinically diagnosed and defined by LTx clinic spirometry, and treated according to established practice and standard protocols at the time (14).

### Pulmonary Toxicity

The onset of EVE induced pulmonary toxicity was diagnosed with the presence of clinical symptoms (e.g., dyspnoea, cough, or fever) and radiological signs of pulmonary involvement (pulmonary computed tomography scans or abnormal chest X-ray) not compatible with other diagnoses. Pulmonary symptoms would typically resolve after the discontinuation of EVE. Although histological diagnosis is considered the gold standard, this was not always undertaken (23). Without radiological findings or the exclusion of other pulmonary diseases, LTR were classified as suspected CLAD according to ISHLT criteria (24).

### General Management Strategy for Renal Preservation

Induction therapy with the IL-2 receptor blocker, basiliximab was given as a CNI-sparing agent to LTR who were identified pre-transplant as being at higher risk of developing post-LTx renal dysfunction (*n* = 95). Subsequent strategies for LTR with renal impairment involved CNI reduction or elimination; control of hypertension, diabetes mellitus, and cholesterol; and initiation of EVE (25). Recipients of both strategies were routinely screened for evidence of proteinuria prior to conversion to EVE based immunosuppression. Proteinuria was not present in any LTR at baseline.

### Study End Points

The primary endpoint for efficacy was survival, with secondary endpoints measured being incidence of CLAD and ACR. An additional secondary endpoint investigated was the impact of discontinuation on survival. The safety of the various strategies was assessed by measuring renal, hematological, and metabolic markers at baseline and 3 months after initiating EVE.

### Statistical Analyses

Continuous data were summarized using means and standard deviations (SD) or medians and interquartile ranges (IQR) depending on distribution, and categorical data as counts and percentages. Comparisons between groups (minimization versus elimination strategy) were made using Student’s t-test or Mann-Whitney U test as appropriate for continuous variables and chi-square or Fisher’s exact test for categorical variables. Overall survival was defined as the time from the date of starting EVE to the date of death or last follow-up.

Univariate and multivariate analyses for overall survival were performed using Cox proportional hazards regression with results reported as hazard ratios (HR) and 95% confidence intervals (95% CI). Variables with a *p* < 0.05 on univariate analyses or those deemed clinically relevant were considered for inclusion in the multivariable models. The Kaplan-Meier product-limit method was used to plot survival as a function of time after starting EVE, and comparisons between curves were made with the log-rank test. Changes in serum creatinine concentrations and eGFR from time of starting EVE to 3 months post were determined using paired t-test or Wilcoxon signed rank test as appropriate. All calculated *p* values were 2-tailed and a *p* < 0.05 indicated statistical significance.

Statistical analyses were performed with SAS version 9.4 (SAS Institute, Cary, NC, United States).

## Results

### Patient Characteristics and Indications for EVE Use

The final cohort consisted of 217 LTR who started on EVE (120 CNI minimization and 97 CNI elimination). Baseline demographics are shown in Table 1. The most common indication for starting EVE was renal preservation (75%), followed by malignancy (13%) and neurotoxicity (8%) (Table 1). The median time from LTx to EVE initiation was 528 days [IQR: 240–1,460] with the median time of follow up for all LTR included being 1998 days [IQR: 938–3,770].

**TABLE 1:** Demographics

Characteristic	CNI minimization (*n* = 120)	CNI elimination (*n* = 97)	*p*-value
Age (yr), mean ± SD	52.8 ± 13.7	49.8 ± 15.0	0.12
Gender: male, (n%)	63 (52.5)	63 (64.9%)	0.07
Indications for transplantation, n (%)			
Chronic obstructive pulmonary disease	50 (41.7)	42 (43.3)	0.32
Cystic fibrosis/bronchiectasis	23 (19.2)	24 (24.7)	0.81
Interstitial lung disease	32 (26.6)	20 (20.6)	0.30
Pulmonary hypertension	9 (7.5)	6 (6.2)	0.70
Redo transplant	6 (5.0)	5 (5.2)	0.96
Transplantation type, n (%)			
Bilateral sequential lung	99 (82.5)	72 (74.2)	0.14
Single lung	16 (13.3)	19 (19.6)	0.21
Heart and lung	5 (4.2)	6 (6.2)	0.50
Early initiation of everolimus^a^	43 (35.7%)	33 (34%)	0.78
Indication for everolimus, (n, %)			
Renal Preservation (163)	87 (72.5)	76 (81.4)	0.32
Malignancy (28)		
Skin cancers	18 (15.0)	6 (6.2)	0.04
Other (PTLD, lung cancer)	1 (0.8)	3 (3.1)
Neurotoxicity (18)			0.48
PRES	0 (0)	4 (4.1)	
Seizures	0 (0)	3 (3.1)	
Neurocognitive disorder	2 (1.7)	2 (2.1)	
Neuropathy (peripheral and optic)	2 (1.7)	1 (1.0)	
Tremor	3 (2.5)	0 (0)	
Migraines	1 (0.8)	0 (0)	
Other (8)			0.91^b^
Augment immunosuppression (6)	5 (4.2)	1 (1.0)	
Recurrent CMV (2)	1 (0.8)	1 (1.0)	

^a^ Everolimus started less than 1 year after lung transplant.

^b^ Other includes other non-skin cancer malignancy.

Abbreviations: CMV, *Cytomegalovirus;* PTLD, post-transplant lymphoproliferative disorder; PRES, posterior reversible encephalopathy syndrome.

#### Efficacy

##### Overall Survival, CLAD and ACR

**FIGURE 1:**
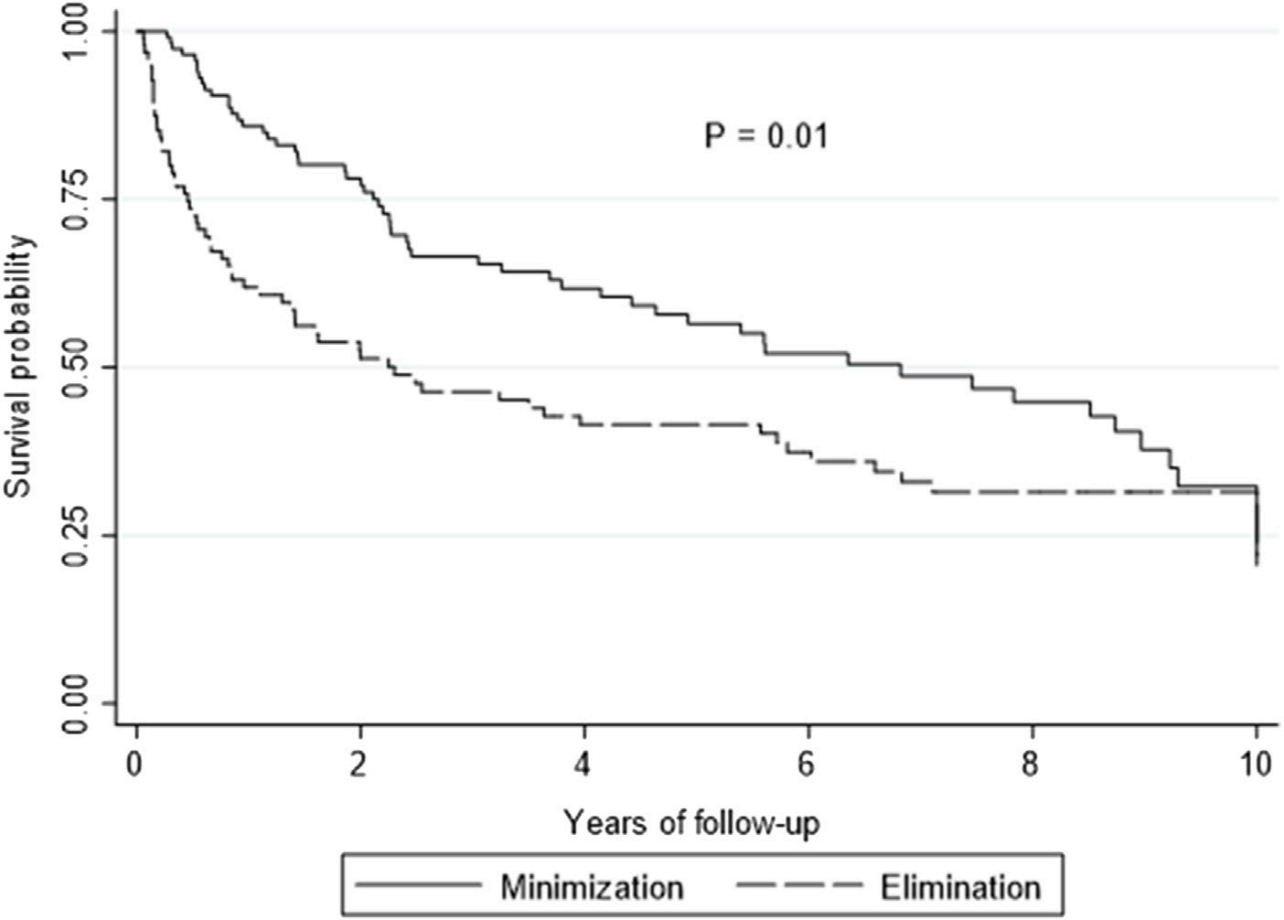
Kaplan-Meier curve showing overall survival by elimination versus minimization. *p*-value is calculated from a log rank test comparing the entire survival experience between the two groups (minimization versus elimination).

On multivariate analysis, LTR who received EVE as part of the CNI elimination strategy had poorer survival outcomes compared to the CNI minimization strategy [HR 1.61, 95% CI: 1.11–2.32, *p* = 0.010] (Figure 1). The median survival for the entire cohort was 1,797 days [IQR: 401–4,427] (CNI minimization: 2,491 days [IQR: 789–4,171] vs. CNI elimination: 840 days [IQR: 166–4,427]). Overall survival between the minimization and elimination groups at 1, 3 and 5 years was significant (1 year: 85.9% vs. 61.9%, *p* ≤ 0.0001, 3 years: 66.5% vs. 46.4%, *p* = 0.004, 5 years: 56.5% vs. 41.5%, *p* = 0.41). The incidence of ACR and CLAD was similar between the groups (*p* = .76 and *p* = 0.83, respectively) (Table 2).

**TABLE 2:** Clinical characteristics: Immunosuppression, theraputic drug monitoring, rejection and comorbidities.

Characteristic (n, %)	CNI minimization (*n* = 120)	CNI elimination (*n* = 97)	*p*-value
Immunosuppression			
Induction, n (%)			
Basiliximab	50 (41.7)	45 (46.4)	0.49
Maintenance Immunosuppression, n (%)^a^			
Tacrolimus	108 (90.0)	73 (75.3)	0.004
Ciclosporin	12 (10.0)	24 (24.7)	0.004
Mycophenolate	31 (25.8)	39 (40.2)	0.024
Azathioprine	33 (27.5)	27 (27.8)	0.96
No Antimetabolite	56 (47.6)	31 (32.0)	0.028
Therapeutic drug monitoring			
Tacrolimus			
Mean tacrolimus dose^b^	4.46 ± 2.94 mg		
Mean tacrolimus level^c^	5.46 ± 1.87 ng/mL		
Ciclosporin			
Mean ciclosporin dose^b^	120.84 ± 45 mg		
Mean ciclosporin concentration^c^	89.91 ± 63.62 ng/mL		
Everolimus			
Mean everolimus dose^b^	1.71 ± 0.91 mg	1.68 ± 1.11 mg	0.87
Mean everolimus concentration^c^	4.14 ± 2.12 ng/mL	5.58 ± 2.59 ng/mL	<0.0001
Rejection			
Rejection, n (%)			
Diagnosis of CLAD^d^	85 (70.8)	70 (72.2)	0.83
Acute Rejection—Pulse of Steroids^e^	18 (15.0)	16 (16.5)	0.76
Antithymocyte globulin (Equine or rabbit)	27 (22.5)	13 (13.4)	0.09
Laboratory results and comorbidities			
Hemoglobin^f^, (mean ± SD)	115.8 ± 17.0 g/L	112.2 ± 21.4 g/L	0.07
White cell count^f^, (mean ± SD)	6.77 ± 2.57 × 10^9^/L	7.68 ± 3.63 10^9^/L	0.032
Platelets^f^, (mean ± SD)	236 ± 76.8 × 10^9^/L	255 ± 123 × 10^9^/L	0.16
Urinary protein^f^ (median, IQR)	0.075 g/L (IQR: 0.04–0.165)	0.16 g/L (IQR: 0.06–0.31)	0.001
Hypertension^g^	74 (61.7)	56 (57.7)	0.56
Diabetes Mellitus^g^	47 (39.2)	37 (38.1)	0.88
Hyperlipidemia^g^	53 (44.2)	41 (42.3)	0.78
*Cytomegalovirus* reactivation^h^	30 (25.0)	32 (32.9)	0.20
*Aspergillus* colonization^h^	43 (35.8)	32 (33.0)	0.66

^a^ At the time of starting EVE.

^b^ Total daily dose within 3 months after initiating.

^c^ Within first 3 months after initiating.

^d^ Diagnosis of CLAD pre or post starting on everolimus.

^e^ Episode of ISHLT graded ≥2 ACR pre or post starting on everolimus.

^f^ Measured at 3 months after starting everolimus.

^g^ Comorbidity at 3 months after starting everolimus.

^h^ CMV Reactivation or *Aspergillus* colonization at any time point after starting everolimus.

Abbreviations: ACR, acute cellular rejection; CLAD, chronic lung allograft dysfunction; CMV, *Cytomegalovirus*; ISHLT, International Society of Heart and Lung Transplantation.

##### Preservation of Renal Function

**FIGURE 2:**
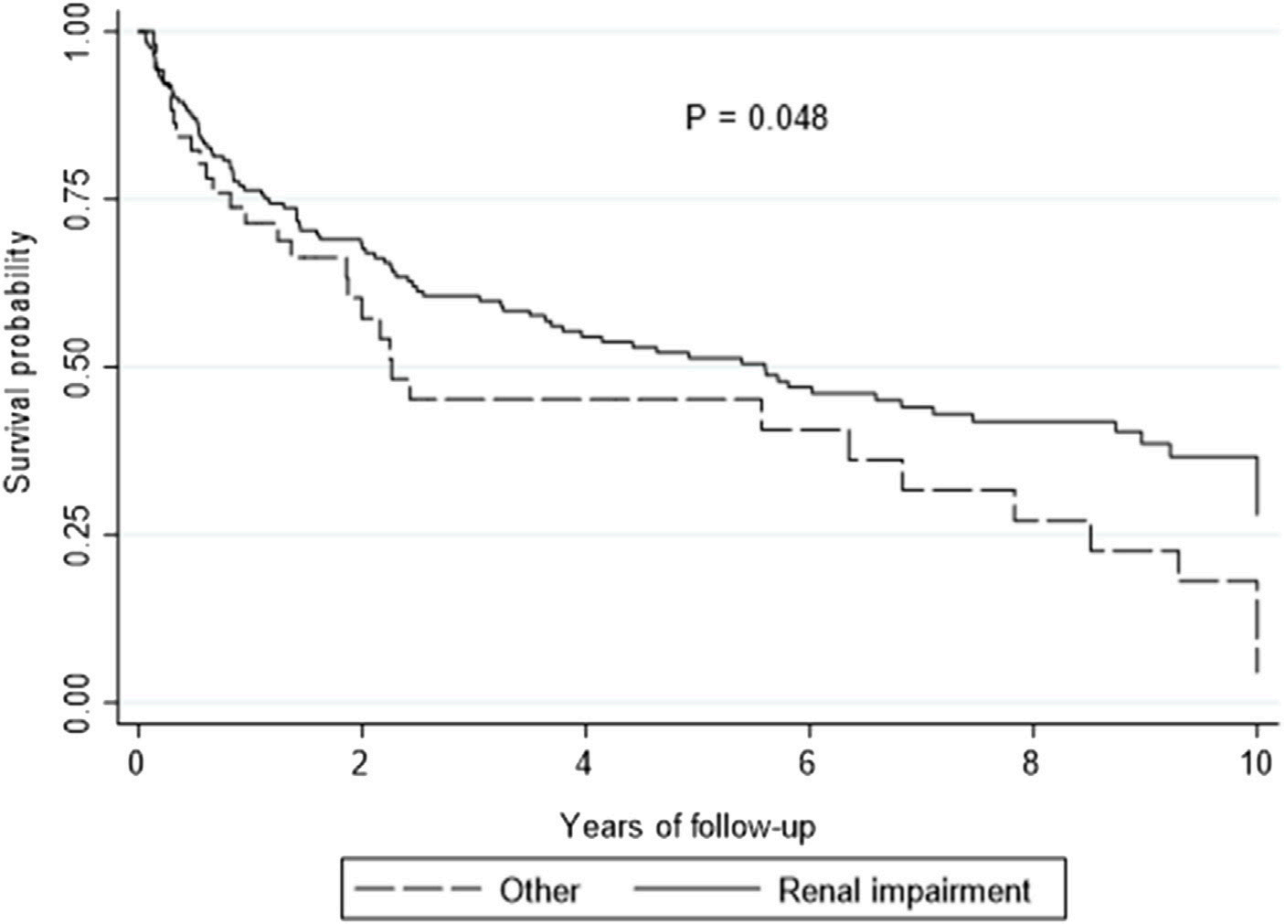
Kaplan-Meier curve showing overall survival by renal preservation versus other indications.

On multivariate analysis, utilization of EVE for renal preservation was associated with improved survival compared to other indications [HR 0.64, 95% CI: 0.42–0.97, *p* = 0.032] (Figure 2). All LTR who started on EVE for renal preservation had an improvement in estimated glomerular filtration rate (eGFR) from 40 ± 16 mL/min/1.73 m^2^ to 47 ± 19 mL/min/1.73 m^2^ (*p* = 0.021) at 3 months after starting EVE.

**FIGURE 3:**
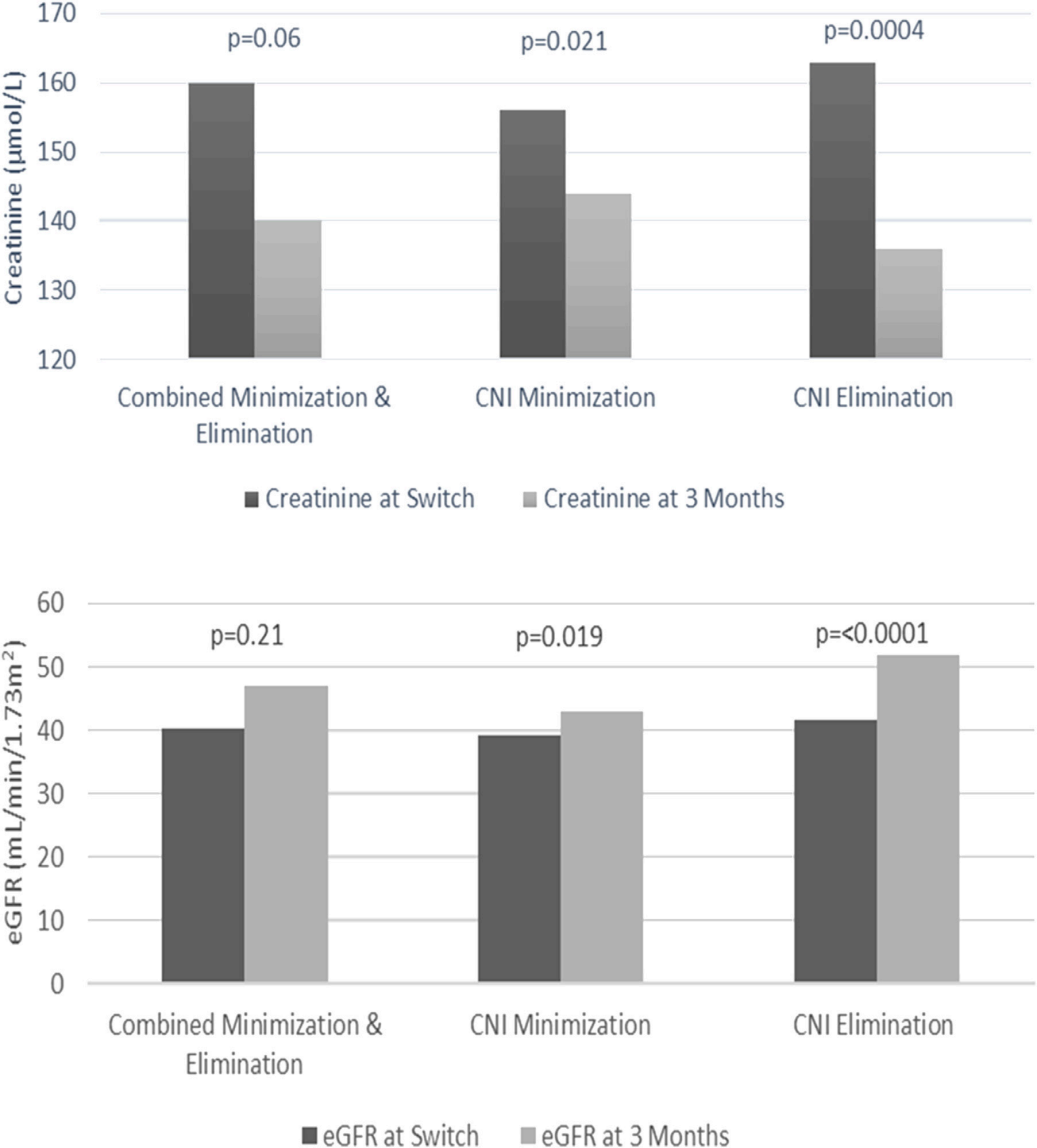
Creatinine and estimated glomerular filtration rate (eGFR) changes from time to switch to 3 months.

The most significant improvements with renal function were demonstrated with the CNI elimination strategy at 3 months after commencing EVE. The mean serum creatinine concentration decreased from 164 ± 63 μmol/L to 136 ± 72 μmol/L (*p* = 0.0004) and eGFR improved from 42 mL/min/1.73 m^2^ to 52 mL/min/1.73 m^2^ (*p* ≤ 0.0001). The improvements over 3 months for measured serum creatinine concentration and eGFR with the CNI minimization strategy were lower but also statistically significant (*p* = 0.021 and *p* = 0.019, respectively) (Figure 3). At 1-year after commencing EVE, measurements of kidney function (creatinine and eGFR) were comparable to those measured at 3 months after starting EVE (139 ± 67 μmol/L and 50 mL/min/1.73 m^2^, respectively).

On multivariate analysis, high urinary protein levels measured at 3 months after starting EVE were associated with poorer survival outcomes [HR 1.21, 95% CI: 1.01–1.45, *p* = 0.033]. Proteinuria in the nephrotic range (≥3,000 mg/24 h) was present in four patients at 3 months after starting EVE, all occurring with the elimination strategy. At 3 months post conversion to EVE, the elimination strategy had a statistically higher level of measured urinary protein compared to the minimization strategy (CNI minimization: 0.075 g/L [IQR: 0.04–0.165] vs. CNI elimination: 0.16 g/L [IQR: 0.06–0.31], *p* = 0.001).

##### Malignancy

The survival outcomes for LTR prescribed EVE for malignancy were comparable to other indications (*p* = 0.631). A statistically higher proportion of LTR received EVE as part of the CNI minimization strategy for malignancy (*p* = 0.04), primarily for the management of skin cancers. EVE was introduced further from LTx (1,358 days [IQR: 743–1,967]), with EVE replacing azathioprine in 62.5% of LTR with malignancy.

##### Neurotoxicity

On univariate analysis, LTR who received EVE in the setting of neurotoxicity had poorer survival outcomes compared to other indications for EVE [HR 1.83, 95% CI: 1.05–3.18, *p* = 0.030], however this was not borne out in multivariate analysis. The median survival for LTR prescribed EVE for neurotoxicity as part of a CNI elimination strategy was markedly lower than the minimization strategy (CNI elimination: 292 days [IQR: 226–652] vs. CNI minimization: 1,725 days [IQR: 696–3,460]).

#### Safety

##### Discontinuation

Discontinuation of EVE due to adverse events was 39.2% for all LTR included in the study with a statistically higher number of LTR discontinuing with the minimization cohort (CNI minimization: 48.3% versus CNI elimination: 29.9%, *p* = 0.002). On univariate analysis, LTR who discontinued EVE did not have poorer survival outcomes compared to those that remained on EVE [HR 1.06, 95% CI: 0.73–1.52, *p* = 0.764]. Pulmonary problems (18.4%), wound healing (5.1%) and edema (4.1%) were the most frequent causes of discontinuation (Table 3).

**TABLE 3:** Everolimus discontinuation.

	CNI minimization	CNI elimination	*p*-value
(n, %)	*n* = 58 (48.3)	*n* = 27 (27.8)	0.002
Pulmonary (40)			
Drop in lung function/CLAD	18 (31)	5 (18.5)	0.23
Pulmonary toxicity	11 (19)	6 (22.2)	0.73
Peripheral oedema (9)	5 (8.6)	4 (14.8)	0.39
Hematological (7)	4 (6.9)	3 (11.1)	0.51
Wound healing (11)	7 (12.1)	4 (14.8)	0.72
Renal (9)			
Proteinuria	3 (5.2)	3 (11.1)	0.38
Dialysis/renal failure	2 (3.4)	1 (3.7)	1.00
Other (9)			
Dermatological (3)	2 (3.4)	1 (3.7)	1.00
Mouth ulcers (2)	2 (3.4)	0 (0)	1.00
Resolution of neurotoxicity (2)	2 (3.4)	0 (0)	1.00
Chronic Infection (1)	1 (1.7)	0 (0)	1.00
Leg pain (1)	1 (1.7)	0 (0)	1.00

Abbreviations: CLAD, chronic lung allograft dysfunction.

##### Pulmonary Toxicity

The most common reason for discontinuation of EVE was pulmonary related, with 18.4% of all patients discontinuing due to pulmonary toxicity or accelerated decline in spirometry/CLAD. LTR with pulmonary toxicity were more likely to discontinue EVE within the first year of initiating EVE (3-month discontinuation, 17.6% and 12-month discontinuation, 82.3%), with the median time of onset to pulmonary toxicity being 154 days [IQR: 107–304].

EVE trough concentrations were not supratherapeutic in LTR with pulmonary toxicity, with the mean trough concentration being 4.07 ± 1.52 ng/mL. All LTR had a full clinical recovery within 1 year of discontinuation with no fatalities attributed to pulmonary toxicity. Thirteen of the seventeen patients with pulmonary toxicity had confirmed CLAD at the time, confounding the diagnosis.

##### Adverse Events and Laboratory Results

The incidence of adverse events was similar between the two treatment groups. Rates of hypertension, new-onset diabetes mellitus and hyperlipidemia were comparable at 3 months after starting EVE. The incidence of CMV reactivation or *Aspergillus* colonization was similar between the groups (Table 2).

Measured hemoglobin concentrations and platelet counts were comparable at 3 months after starting on EVE, whereas the CNI minimization group accounted for a statistically lower white cell count (*p* = 0.032) (Table 2).

##### Immunosuppression and Therapeutic Drug Monitoring

The most common immunosuppression regimens were EVE, tacrolimus, and prednisolone for CNI minimization (42.5%) and EVE, mycophenolate, and prednisolone for CNI elimination (40.2%). As expected, the mean EVE trough concentration within the first month after starting EVE was significantly lower with CNI minimization (mean trough concentration: 4.14 ± 2.12 ng/mL [Aim 3–5 ng/mL]) compared to CNI elimination (mean trough concentration: 5.58 ± 2.59 ng/mL [Aim 5–7 ng/mL], *p* ≤ 0.0001). Key differences in immunosuppressive strategies are outlined in Table 2. Weaning dosage protocols after LTx for antimetabolite and prednisolone after LTx were comparable across the study groups.

On multivariate analysis, immunosuppressant regimens containing mycophenolate were associated with improved survival [HR 0.66, 95% CI: 0.44–0.99, *p* = 0.038]. All other immunosuppression and serum trough levels within the first month after starting EVE had no impact on survival (Table 4).

**TABLE 4:** Univariate and multivariate analysis of factors influencing survival.

Summary of effects of different covariates on survival			
	Variable	Hazard ratio (95% CI)	*p*-value
Univariate Analysis			
Demographics	Male	0.88 (0.61–1.26)	0.468
	Age	1.01 (0.99–1.02)	0.446
Everolimus Strategy	Early initiation of everolimus^a^	1.13 (0.77–1.67)	0.524
	Elimination strategy	1.54 (1.07–2.22)	0.017
Rejection	CLAD diagnosis^b^	1.83 (1.17–2.86)	0.007
Indication for Everolimus	Renal Preservation	0.64 (0.42–0.98)	0.034
	Neurotoxicity	1.83 (1.05–3.18)	0.030
	Skin cancer	0.83 (0.38–1.82)	0.631
Renal Indicators	Creatinine (Introduction of everolimus)	1.00 (1.00–1.01)	0.358
	Creatinine (3 months after introduction)	1.00 (1.00–1.00)	0.314
	Urinary protein	1.22 (1.03–1.46)	0.021
Immunosuppression	Basiliximab	1.31 (0.90–1.89)	0.148
	Tacrolimus	1.40 (0.87–2.25)	0.162
	Ciclosporin	0.72 (0.44–1.15)	0.162
	Mycophenolate	0.66 (0.45–0.99)	0.040
	Azathioprine	1.05 (0.71–1.55)	0.804
	No antimetabolite	1.46 (0.99–2.14)	0.051
Therapeutic Drug Monitoring^c^	Tacrolimus level^d^	1.07 (0.75–1.54)	0.704
	Everolimus level	1.01 (0.94–1.07)	0.863
Multivariate analysis			
Variable	Elimination strategy	1.61 (1.11–2.32)	0.010
	Renal preservation	0.64 (0.42–0.97)	0.032
	Urinary protein	1.21 (1.01–1.45)	0.033
	Mycophenolate	0.66 (0.44–0.99)	0.038

^a^ Everolimus started less than 1 year after lung transplant.

^b^ Chronic lung allograft dysfunction pre or post starting everolimus.

^c^ Average level within the first 3 months after starting everolimus.

^d^ For minimization strategy.

Abbreviations: ACR, acute cellular rejection; CLAD, chronic lung allograft dysfunction.

##### Cause of Death

There was no difference in CLAD-related mortality between the two cohorts (25.0% CNI minimization vs. 27.8% CNI elimination, *p* = 0.36). A statistically higher number of LTR died from infection within the elimination group (*p* = 0.036). Sepsis (bacterial *n* = 8, fungal *n* = 1), viral pneumonia (*n* = 1) and CMV infection (*n* = 1) were the most common reasons for infection-related mortality. Although the most common indication for starting EVE, death due to renal failure occurred in only six patients (Table 5).

**TABLE 5:** Cause of death.

Cause of death	CNI minimization	CNI elimination	*p*-value
(n, %)	*n* = 57 (47.5)	*n* = 61 (62.8)	0.024
CLAD	30 (25.0)	27 (27.8)	0.36
NSGF	11 (9.2)	9 (9.3)	0.51
Cancer	10 (8.3)	6 (6.2)	0.22
Infection	2 (1.7)	9 (9.3)	0.036
Renal failure	3 (2.5)	3 (3.1)	0.93
Coronary vascular accident	1 (0.8)	4 (4.1)	0.20
Other	0 (0)	3 (3.1)	0.09
Multi-organ failure			
Pancreatitis			
Hemophagocytic lymphohistiocytosis			

Abbreviations: CLAD, chronic lung allograft dysfunction; NSGF, non-specific graft failure.

## Discussion

### Minimization Versus Elimination

We believe that this is the largest study investigating the feasibility of CNI-free maintenance regimens. Our study found that LTR receiving EVE as part of an elimination strategy had poorer survival outcomes compared to the minimization strategy. A potential explanation could be that the higher mortality rate for the elimination strategy reflects the clinical complexity of this group requiring complete CNI withdrawal. The elimination strategy included LTR with a higher measured serum creatinine, advanced malignancy, sepsis, or manifestations of neurotoxicity, such as seizures or PRES. Compared to ISHLT registry data, the 1 and 5-year survival outcomes for both groups receiving EVE was markedly lower (26), indicating that the selection of our LTR receiving EVE may be representative of a clinically complex group of LTR. The incidence of CLAD between the two groups was also higher compared to ISHLT registry data, with CLAD being the leading cause of death for both strategies (27).

Nonetheless, CNIs remain the cornerstone of maintenance immunosuppressive regimens and our study provides further evidence that inclusion of CNIs remain paramount and long-term withdrawal may adversely impact survival. Considering the poorer survival outcomes for the elimination strategy, an assessment of the risks for a rechallenge with a lower dose CNI should be undertaken once stabilization of the LTR occurs, especially for those early after LTx. An alternative could be the incorporation of other CNI-sparing agents with EVE-based immunosuppression, such as intravenous immunoglobulin (IVIG) or belatacept to elevate survival outcomes to those comparable with CNI-based immunosuppression. This approach would require further investigation.

#### Efficacy

##### Preservation of Renal Function

Renal impairment remains challenging post LTx, especially in the perioperative period with an increased risk of LTx-related morbidity and mortality (28). Management of renal impairment with EVE after LTx has been undertaken in several other studies (11, 13, 29, 30), and we demonstrated similar improvements with renal function, with no significant renal related mortality. We found that initiating EVE for renal preservation had superior survival outcomes compared to other indications (Figure 3).

Although CNI elimination was the most effective strategy at improving renal function, this was offset by the poorer survival outcomes compared to CNI minimization group. The benefits of the CNI elimination strategy on renal function need to be balanced against the risks of having a CNI-free regimen on survival. Based on the findings of our study, CNI minimization with close monitoring of creatinine would be the suggested approach when starting EVE for renal preservation. If creatinine continues to deteriorate, then temporary transition to the elimination strategy with future rechallenge with low dose CNI or the introduction with alternative immunosuppressants may be required.

##### Malignancy

Existing transplant literature has postulated that transitioning from a CNI to EVE may have beneficial effects in LTR with malignancy, due to its effect on cell metabolism and proliferation (31, 32). Our cohort of LTR were typically initiated with EVE for malignancy further from LTx, highlighting the long-term impact of immunosuppression on the emergence of skin malignancies. Although malignancy represented a relatively small cohort with the majority being non-melanoma skin cancers, the survival outcomes were successful with comparable survival to other cohorts with only two deaths from skin malignancy. CNI minimization appears to be the preferred immunosuppressive strategy for LTR with skin malignancies. Cessation of the antimetabolite should be undertaken pre-emptively at the earliest sign of skin malignancy for high risk LTR.

##### Neurotoxicity

CNI-induced neurotoxicity can manifest in severity from headaches or tremor to life-threatening complications such as seizures or posterior reversible encephalopathy syndrome (PRES). The transplant literature notes early major neurologic complications post LTx can result in substantive morbidity and mortality (33–35), and our center experienced similar challenges and outcomes.

On univariate analysis, LTR who received EVE for the management of neurotoxicity had the poorest survival outcomes. Most noteworthy were LTR with neurotoxicity allocated to the CNI elimination group with a median survival of only 292 days from the date of commencing EVE. Although unavoidable in some settings, our findings support the evidence that early CNI withdrawal is not recommended. Less severe manifestations of neurotoxicity, such as tremor or headaches may be resolved with the CNI minimization strategy, where improvement of symptoms may occur with lower target CNI target levels.

#### Safety

##### Discontinuation of Everolimus

The rate of discontinuation of EVE for our cohort was 39.6%, and these rates are consistent with other LTx studies ranging in incidence from 25%–55% (9–11). We hypothesized that high rates of discontinuation of EVE documented elsewhere are potentially destabilizing for immunosuppressant regimens with poorer survival outcomes. Our study found that the high rates of discontinuation of EVE did not contribute to poorer survival outcomes. A potential explanation is that alternative immunosuppressants would typically be substituted in LTR discontinuing EVE, lessening periods of subtherapeutic immunosuppression, and improving survival outcomes.

##### Impact on Pulmonary Function

One of the most serious side effects from EVE is pneumonitis that can result in life-threatening complications (36). EVE-induced pneumonitis has been limited to case reports after thoracic transplantation (7), and our study identified 17 potential cases, corresponding with an incidence of 8.2%. Most cases of pulmonary toxicity occurred within the first year, consistent with the available literature (37). Although higher trough concentrations of EVE are associated with pulmonary toxicity (38), we found that with our cohort all had serum trough concentrations within the target range.

Unsurprisingly, all LTR had underlying pulmonary disease making it difficult to distinguish CLAD from pulmonary toxicity. LTR with pulmonary toxicity all had radiological changes, confirming organizing pneumonia or interstitial pneumonitis. A limitation of our study was that histopathological diagnosis was not undertaken in all suspected cases.

##### Adverse Events

Side effects from EVE reported in other studies in LTx remain high, ranging from 35%–82% (9, 10, 40). Cardiovascular risk factors, such as hyperlipidemia, hypertension and diabetes are all associated with immunosuppression regimens (5, 41). At 3 months after starting an EVE based regimen, we found no significant difference in the incidence of the cardiovascular risk factors. Hematology parameters suggested that bone marrow toxicity was not a safety concern with EVE for either regimen.

The most common non-pulmonary related reason for discontinuing was edema. Incidence of edema leading to discontinuation was consistent with other published data (42). Similarly, we found that patients who ceased due to edema tended to be earlier post LTx.

##### Immunosuppression and Therapeutic Drug Monitoring

Our study highlighted the key differences between the two groups and provided an insight into our immunosuppression strategies. The most significant finding was that mycophenolate containing immunosuppressant regimens were associated with improved survival. Given the potential survival benefits of mycophenolate, we would suggest including mycophenolate in regimens, especially in those where the CNI has been withdrawn.

Therapeutic drug monitoring of EVE after LTx is not well defined. Transplant literature has recommended a serum trough concentration (C_(0)_ of 3–8 ng/mL or 3–12 ng/mL has been postulated as part of minimization regimens post LTx (13, 15). Our study demonstrated lower EVE concentration targets compared to the existing transplant literature. Considering the lagging survival outcomes, it would appear there is a potential to raise the serum trough concentrations of EVE for CNI minimization. However, this approach would need to proceed with caution as it could potentially increase the risk of further intolerance in a cohort with high rates of discontinuation. In addition, this may not be due to a dose-dependent effect as other underlying disease mechanisms may not be targeted by our current agents.

## Limitations

There are several limitations to this study. Firstly, the study is retrospective with all data obtained from chart reviews. Additionally, the findings reflect a single center experience, our institution did not have a detailed protocol to guide usage, and usage has also evolved over time. Our study was non-randomized, and the cohort was heterogenous, with multiple indications for EVE being included in the two cohorts. Allocation to either strategy was dependent on clinical judgement at the time of starting on EVE. Although demographics between the two groups were similar, CNI withdrawal was more common in those acutely unwell potentially biasing the outcomes. Noting the difficulty of prospective randomized controlled studies, our study did not compare the survival outcomes for CNI minimization or elimination strategies against standard CNI based immunosuppressive regimens. Future studies could potentially investigate this approach.

## Conclusion

EVE can be successfully utilized for maintenance immunosuppression post lung transplant, particularly where the indication is for renal preservation. However, immunosuppressive regimens containing some CNI had superior survival outcomes, highlighting the importance of retaining a CNI wherever possible. Future studies could potentially investigate the impact of lower CNI target levels in those with demonstrated previous intolerance and the subsequent impact on survival.

## Data Availability

The raw data supporting the conclusion of this article will be made available by the authors, without undue reservation.
